# Phosphatidic acid: biosynthesis, pharmacokinetics, mechanisms of action and effect on strength and body composition in resistance-trained individuals

**DOI:** 10.1186/s12986-017-0166-6

**Published:** 2017-02-06

**Authors:** Peter Bond

**Affiliations:** PeterBond.nl, Waterhoenlaan 25, 3704 GV Zeist, The Netherlands

**Keywords:** Phosphatidic acid, mTORC1, Muscle hypertrophy

## Abstract

The mechanistic target of rapamycin complex 1 (mTORC1) has received much attention in the field of exercise physiology as a master regulator of skeletal muscle hypertrophy. The multiprotein complex is regulated by various signals such as growth factors, energy status, amino acids and mechanical stimuli. Importantly, the glycerophospholipid phosphatidic acid (PA) appears to play an important role in mTORC1 activation by mechanical stimulation. PA has been shown to modulate mTOR activity by direct binding to its FKBP12-rapamycin binding domain. Additionally, it has been suggested that exogenous PA activates mTORC1 via extracellular conversion to lysophosphatidic acid and subsequent binding to endothelial differentiation gene receptors on the cell surface. Recent trials have therefore evaluated the effects of PA supplementation in resistance-trained individuals on strength and body composition. As research in this field is rapidly evolving, this review attempts to provide a comprehensive overview of its biosynthesis, pharmacokinetics, mechanisms of action and effect on strength and body composition in resistance-trained individuals.

## Background

Skeletal muscle mass comprises roughly half of our body mass and is essential for locomotion, heat production during periods of cold stress and overall metabolism [1]. Skeletal muscle mass can be increased by mechanical loading such as a resistance exercise program [2]. The hypertrophic response to mechanical loading can be enhanced by employing dietary strategies, such as optimizing protein intake [3], and supplementation strategies, such as creatine monohydrate provision [4]. At the cellular level, mechanical loading, protein intake, and several sports supplements have been found to regulate mechanistic target of rapamycin complex 1 (mTORC1) activity [5]. mTORC1 is a protein complex consisting out of the three core subunits mTOR, Raptor and mLST8 [6]. mTOR forms the catalytic center of the complex and functions as a serine/threonine protein kinase belonging to the phosphatidylinositol-3 kinase (PI3K)-related kinase (PIKK) superfamily [7]. mTORC1 acts as a signal integrator of various environmental cues and controls protein synthesis, specifically the process of protein translation initiation, through its downstream effectors p70 ribosomal protein S6 kinase 1 (p70S6K1) and eukaryotic translation initiation factor 4E-binding protein 1 (4E-BP1) [8]. Phosphorylation, and thereby activation, of p70S6K1 modulates functions of translation initiation factors [9] and might also promote ribosome biogenesis and resultingly increase the translational capacity of the cell [10]. Its other substrate, 4E-BP1, inhibits mRNA translation initiation by preventing formation of the eIF4F complex which facilitates recruitment of the small (40S) ribosomal subunit to the 5′ end of mRNA [11]. Phosphorylation of 4E-BP1 by mTORC1 results in its dissociation of the mRNA strand and therefore relieves the inhibition it poses on formation of the eIF4F complex.

Several inputs which regulate mTORC1 have been identified, such as growth factors (e.g. insulin [12] and insulin-like growth factor 1 [IGF-I] [13]), amino acids [14], mechanical stimuli [15] and energy status [16]. Interestingly, regulation of mTORC1 activity by mechanical stimuli has been suggested to be mediated by phosphatidic acid (PA) formation [17]. Moreover, the branched-chain amino acid leucine, an important regulator of mTORC1 activity, has also been found to activate phospholipase D1 (PLD1) and induce its subcellular translocation to the lysosome (the site of mTORC1 activity) [18]. PLD1 hydrolyses phosphatidylcholine (PC) producing PA.

PA is a phospholipid consisting out of a glycerol backbone with two fatty acids and one phosphate group attached to it. The two fatty acids are attached to two neighboring C-atoms at position *sn*-1 and *sn*-2, with the phosphate group attached to the C-atom at position *sn*-3. The fatty acid at the *sn*-1 position is often a saturated one, whereas the fatty acid at the *sn*-2 position is often unsaturated [19]. The fatty acid composition of PA appears critical in its ability to activate mTORC1. Specifically, research has shown that PA species containing one or two unsaturated fatty acid chains activate mTORC1 both in human embryonic kidney (HEK) 293 cells, as well as in vitro, whereas saturated PA species have no significant effect [20]. Furthermore, in a comparison between soy-derived PA and egg-derived PA, soy-derived PA was more effective in increasing mTORC1 signaling, as derived from p70S6K1 phosphorylation on Thr389, in C2C12 myoblasts [21]. It is appealing to speculate that the higher unsaturated fatty acid content of soy-derived PA compared to egg-derived PA underlies this difference.

Given the apparent role of PA in mTORC1 regulation, researchers soon evaluated its supplementation in resistance-trained men in order to assess its effect on strength, muscle thickness and lean tissue accruement in a pilot study [22]. Following this pilot study, several other human trials have also evaluated the effect of phosphatidic acid supplementation in athletes [21, 23–25]. As research in this field is rapidly evolving, this review attempts to provide a comprehensive overview of its biosynthesis, pharmacokinetics, mechanisms of action and effect on strength and body composition in resistance-trained individuals.

## Biosynthesis and metabolism

**Fig. 1:**
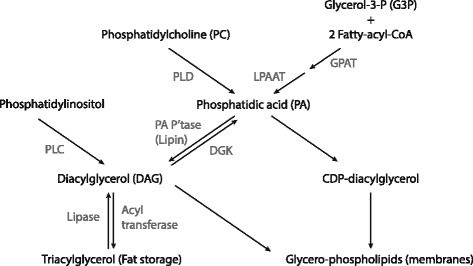
Metabolism of PA. PA can be synthesized from various sources. A *de novo* pathway originates from G3P. G3P is acetylated twice, requiring fatty-acyl-CoA for its acetylation. First it is acetylated by GPAT and then by LPAAT. A second pathway uses PC. PC is hydrolyzed by PLD to produce PA. Finally, PA can be produced by the phosphorylation of DAG by DAG kinase (DGK). DAG is derived from triacylglycerols and phosphatidylinositol. PA phosphatase (PA P’tase) is responsible for dephosphorylation of PA to DAG. Various CDP-diacylglycerol synthases produce CDP-diacylglycerol from PA. Figure based on [27]

PA holds a central role in membrane glycerophospholipid and triacylglycerol synthesis as their biosynthetic precursor [26] and can be generated by three major mechanisms [27] (see Fig. 1). One of these metabolic pathways is able to generate PA *de novo*. This *de novo* pathway originates from glycerol-3-phosphate (G3P). G3P can be formed from one of the intermediate products of glycolysis. During glycolysis sugar is converted into fructose-1, 6-biphosphate and subsequently cleaved into two three-carbon units, namely dihydroxyacetone phosphate (DHAP) and glyceraldehyde-3-phosphate (not to be confused with G3P). The generated DHAP can then be reduced into G3P, a reaction catalyzed by glycerol-3-phosphate dehydrogenase (GDPH). GDPH is an integral membrane protein of the endoplasmic reticulum (ER) and outer mitochondrial membrane and its expression in the outer mitochondrial membrane is induced by insulin signaling [28]. Following the production of G3P, two acylation reactions take place to generate PA. The first acylation reaction producing lysophosphatidic acid (LPA) is catalyzed by glycerol-3-phosphate acyltransferase (GPAT) and the second acylation, resulting in PA, is catalyzed by lysophosphatidic acid acyltransferase (LPAAT). Both dietary fatty acids and *de novo* synthesized palmitic acid can contribute the required acyl-CoA groups. Moreover, overexpression of LPAAT-θ has been found to induce mTOR-dependent p70S6K1 and 4E-BP1 phosphorylation and is expressed in skeletal muscle tissue [29]. Several other isoforms also appear to be expressed in murine skeletal muscle [30], although their role in mTOR regulation is unclear. Currently, not much is known about the regulation of LPAAT by growth factors or nutrients. Finally, it should be noted, that this *de novo* pathway has a strong preference for producing PA species with two saturated fatty acids [31].

In a second pathway, PC is hydrolyzed into PA and choline. A reaction catalyzed by phospholipase D (PLD). PLD has been hypothesized to play a crucial role in the mechanical activation of mTOR signaling and the isozymes PLD1 and PLD2 can be found in the z-band of skeletal muscle [32]. Moreover, pharmacological inhibition of PLD with the primary alcohol 1-butanol prevents both an increase in PA as well as mTOR signaling [32]. However, recent research showed that activation of PLD was not required for the mechanically induced increase in mTOR signaling [33]. This appears to contradict previous experiments which showed that PLD was crucial in mediating this increase. However, these experiments were mainly based on pharmacological inhibition of PLD by 1-butanol. Concerns were raised that some of 1-butanol’s biological effects are not specific to inhibiting PLD activity [34]. Moreover, earlier findings indicated that mechanically induced PLD activity poorly correlated with the cellular increase of PA [32]. PLD1 has also recently been implicated in mTORC1 activation by the branched-chain amino acid leucine [18], an important regulator of mTORC1 activity [35]. It was found that, in a leucine-dependent manner, leucine tRNA synthetase interacts with the lipid kinase Vps34. In turn, Vps34 produces phosphatidylinositol-3-phosphate (PI(3)P) which interacts with the PX domain of PLD1. This interaction translocates PLD1 to the lysosome, the site of mTORC1 activity, and produces PA. Additionally, some growth factors, such as insulin and insulin-like growth factor 1, have also been demonstrated to regulate PLD activity [36].

A third pathway which generates PA utilizes diacylglycerol (DAG) as its substrate. DAG can emanate from stored fat as triacylglycerol, as well as the glycerophospholipid phosphatidylinositol (PI). In order to produce DAG from triacylglycerol, one of the outer acyl-CoA groups is deacylated by a lipase. Conversely, DAG synthesis from PI requires removal of the inositol group. The enzyme phospholipase C (PLC) catalyzes this reaction. DAG is phosphorylated by DAG kinase (DGK) or PRK-like ER kinase (PERK) into PA [37]. PERK is found in the endoplasmic reticulum (ER) membrane. Notably, it exhibits phosphoinositide-3 kinase (PI3K)-dependent kinase activity [37]. This might provide an interesting link between PA-mTORC1 signaling and the canonical activation of mTORC1 by growth factors mediated by PI3K/Akt signaling. Nevertheless, this link currently remains largely unexplored. A larger body of literature has examined the role of DGKs. DGKs belong to a large family of intracellular lipid kinases [38]. Several isoforms have been identified and the ζ-isoform has received special attention as overexpression of the isoform in serum-deprived HEK293 cells lead to an increase in p70S6K1 phosphorylation in an mTOR-dependent manner [39]. Moreover, DGKζ has recently been shown to be necessary for a mechanically induced increase in PA-mTOR signaling [33]. Overexpression of DGKζ was found to be sufficient to induce muscle fiber hypertrophy through an mTOR-dependent mechanism.

In summary, current evidence indicates that PA synthesized by DGKζ, but not PLD, is responsible for the mechanical activation of mTOR signaling and hypertrophy. However, PLD has been implicated in mTORC1 activation by leucine and PLD activity has been demonstrated to be regulated by growth factors such as insulin and IGF-1. The first step in the *de novo* pathway originating from GDP and catalyzed by GPAT, is induced by insulin. However, the regulation of the second and final step by growth factors and nutrients remains unexplored.

The metabolism of PA follows two important paths in the *de novo* synthesis of glycerophospholipids and triacylglycerol. One path leads to the storage of energy in adipose tissue: triacylglycerol biosynthesis. A PA phosphatase hydrolyzes the bond with the phosphate group to yield DAG and P_i_ [40]. The formed DAG can then be esterified with a third fatty acyl-CoA group to produce triacylglycerol for fat storage. The produced DAG can also be diverted towards the Kennedy pathway yielding the two glycerophospholipids phosphatidylethanolamine (PE) and PC [41]. Both form important constituents of mammalian cell membranes, with PC being the most abundant and PE being the second most abundant phospholipid [42].

A second pathway is directly aimed at glycerophospholipid synthesis. PA is activated by cytidine diphosphate (CDP) forming CDP-diacylglycerol and is catalyzed by CDP-diacylglycerol synthase [26]. This step is quite analogous to the activation of glucose by uridine diphophate (UDP) as seen in glycogen synthesis. Following the activation of PA by CDP, it can then be converted to PI, phosphatidylglycerol (PG) and cardiolipin (CL). PI is a precursor of phosphoinositides, such as phosphatidylinositol (3, 4, 5)-trisphosphate (PIP3), which play an important role in intracellular signaling, vesicular trafficking and cytoskeleton dynamics [43]. PG and CL play an important role in proper functioning of the mitochondria [44] and are also involved in molecular signaling of numerous other cellular processes [45].

Additionally, the acyl group at position *sn*-2 can be hydrolyzed by phospholipase A2 (PLA_2_) producing LPA.

## Absorption

Several foodstuffs contain PA, albeit in extremely small quantities. Using a thin-layer-chromatography-imaging technique, Tanaka et al. quantified the PA content in 38 foodstuffs and 3 herbs [46]. The largest amounts of PA were found in vegetables belonging to *Brassicaceae*, such as cabbage (*Brassica oleracea*) which contained 700 nmol/g (approximately 0.5 mg/g). Human trials have employed PA supplements with doses varying from 250 mg [24] to 750 mg [21–23, 25] in resistance-trained men. As such, the amount of PA found in the diet is negligible to what is employed for supplementation purposes in resistance-trained individuals.

When administered orally, PA is metabolized to lysophospholipids and glycerol-3-phosphate in the intestinal lumen by several pancreatic phospholipases [19]. These pancreatic phospholipases hydrolyze the ester bonds at position *sn*-1 or *sn*-2. There appears to be a specificity for the bond at *sn*-2, in particular by PLA_2_ [47]. Subsequently, these products (mainly lysophospholipids with a fatty acid at position *sn*-1) are then absorbed by the intestinal mucosa. The lysophospholipids can then be re-esterified with a fatty acid in the enterocytes, thus producing PA again. Further esterification to form triacylglycerol can also take place. The formed phospholipids will be incorporated into the outer layer of chylomicrons. Following exocytosis, the chylomicrons will be transported through the lymphatic system in order to arrive in the blood circulation. Due to its transport via the lymphatic system, rather than through the liver via the portal vein, it will take longer to reach the circulation than most other sports supplements. A poster presentation reports that, after a single dose of 1.5 g soy-derived PA in one subject, peak plasma concentrations of PA are reached at 3 h after oral ingestion and were still elevated after 7 h [48]. The peak PA concentration was 32% higher than the baseline value of 2.66 nmol/ml. The baseline plasma concentration of LPA was 0.11 nmol/ml and it showed a bimodal absorption kinetic with peaks after 1 h (+500%) and 3 h (+264%), after almost dropping back to baseline after 2 h. Nevertheless, data in a single subject is of very limited value. Future research investigating PA pharmacokinetics in multiple subjects with varying doses is warranted.

It is currently unclear how much PA eventually reaches skeletal muscle tissue and is absorbed by the muscle cells or is incorporated in their membranes. However, when added to cell culture media, PA is rapidly incorporated into cellular membranes [49]. Nevertheless, for its action on mTORC1 activity it might not be required for PA to reach skeletal muscle cells intact. Some evidence suggests that exogenous PA must be metabolized extracellularly to LPA in order to activate mTORC1 [50].

## Mechanisms of action

In 2001, Feng et al. demonstrated a key role of PA in mTORC1 regulation [49]. In their experiment, an extracellular concentration of 100 μM PA stimulated p70S6K1 activity and 4E-BP1 phosphorylation in HEK263 cells. Addition of the mTOR inhibitor rapamycin abolished this effect, thus indicating that the stimulation of p70S6K1 activity and 4E-BP1 phosphorylation by PA was mTOR dependent. Still it is unknown if similar extracellular PA concentrations are reached in humans after oral ingestion of PA, although the previously discussed study demonstrated a plasma concentration of only ~3 μM after ingestion of 1.5 g PA. Additionally, serum-starved HEK263 cells stimulated with 10% serum showed an increase in cellular PA, p70S6K1 activity and 4E-BP1 phosphorylation. Treatment with 0.3% 1-butanol inhibited p70S6K1 activity, 4E-BP1 phosphorylation and the increase in PA. These observations lead to the hypothesis that mitogenic stimulation of mTOR is mediated by PA. Later evidence revealed that PA can modulate mTOR activity by direct binding to its FKBP12-rapamycin binding (FRB) domain [51]. The FRB domain lends its name to the potent pharmacological mTOR inhibitor rapamycin which tightly binds the site in complex with FKBP12 and thereby inhibits mTOR catalytic activity. A later experiment by Hornberger et al. demonstrated that mechanical stimulation, which increases the cellular PA concentration, increased the half maximal inhibitory concentration (IC_50_) of rapamycin compared to control. This provided further evidence supporting the hypothesis that competition takes place between the FKBP12-rapamycin complex and PA for binding to the FRB domain [32]. However, since rapamycin must be administered exogenously, this does not explain an effect of PA on mTORC1 activity in the absence of rapamycin. An endogenous inhibitor, FKBP38, also binding to the FRB domain, was later identified [52]. Importantly, Yoon et al. demonstrated that FKBP38 is displaced by PA and thereby alleviates the inhibition it imposes on mTORC1 activity [53]. Additionally, they also determined that PA was able to allosterically activate mTORC1, since PA was still able to stimulate mTORC1 signaling after FKBP38 knock-down. Therefore the authors proposed that the action for PA activation of mTORC1 was twofold by: i) displacing the endogenous inhibitor FKBP38 from the FRB domain, and ii) allosterically stimulating catalytic activity of the complex.

In contrast, it has been suggested that exogenous PA does not activate mTORC1 through internalization and subsequent direct interaction with mTOR. Instead, PA would require extracellular conversion to LPA by phospholipases which would then bind and activate endothelial differentiation gene (EDG-2) receptors on the cell surface [50]. Activation of this G-protein coupled receptor would then activate the MEK-ERK pathway. Activation of this pathway can then stimulate mTORC1 activation by inhibiting the tuberous sclerosis complex [54] and Raptor [55]. In addition to activation of the MEK-ARK pathway, the authors also propose that activation of EDG-2 leads to a rise in intracellular PA due to an increase in PLD activity.

In order to test the hypothesis that a mechanically-induced increase in PA activates mTORC1, You et al. employed the MEK/ERK inhibitor U0126 in an ex-vivo model [56]. Intermittent passive stretch was applied to mouse extensor digitorum longus (EDL) muscles for mechanical stimulation. This resulted in an increase in ERK phosphorylation (Thr202/Tyr204) which indeed was effectively blocked by addition of U0126, thus validating its usage as an inhibitor in their experimental model. However, while U0126 decreased the basal levels of p70S6K1 phosphorylation (Thr389 and Thr421/Ser424), it did not block the mechanically-induced increase in phosphorylation of p70S6K1 on these residues. Similarly, mechanical stimulation increased 4E-BP1 phosphorylation (Ser64) both with and without U0126. These results suggest that ERK is not necessary for mechanically-induced mTOR signaling. Nevertheless, U0126 did attenuate the increase in phosphorylation of both p70S6K1 and 4E-BP1 in response to mechanical stimuli. Additionally, the authors incubated C2C12 myoblasts with exogenous PA in the presence or absence of U0126. This lead to similar results as found with the ex-vivo model. U0126 did not block the increase in phosphorylation of either 4E-BP1 and p70S6K1, but nevertheless attenuated it. Based on the assumption that PA is required for a mechanically-induced activation of mTOR, the observation that PA increased in the ex-vivo model and the similarity in results with the in vitro experiment, the authors suggest that mechanical stimulation induces mTOR signaling via an ERK-independent mechanism that potentially involves PA.

In addition, PA might promote an increase in muscle mass by affecting the expression of various factors involved in the ubiquitin-proteasome pathway. The FoxO family of transcription factors play an important role in muscle protein breakdown by modulating the activity of the ubiquitin-proteasome and autophagy-lysosomal proteolytic pathways [57]. Among the factors regulated by FoxO are the two E3 ligases muscle atrophy F-box (MAFbx, also known as atrogin-1) and muscle ring finger 1 (MuRF1). Both of which are considered important regulators of muscle atrophy [58, 59]. Overexpressing the PA-generating enzyme PLD1 in fully differentiated L6 myotubes decreased FoxO3, MAFbx and MuRF1 mRNA expression [60]. Additionally, incubation of the myotubes with 100 μM PA also inhibited the dexamethasone-induced increase in mRNA expression of these factors. Importantly, addition of exogenous PA was able to attenuate both dexamethasone and tumor necrosis factor alpha (TNFα)-induced atrophy of the myotubes. A possible mechanism for this action of PA might be via mTORC2. mTORC2 differs structurally from mTORC1 in that it not contains Raptor but Rictor [61]. Its role in muscle hypertrophy appears less prominent than that of mTORC1. However, mTORC2 phosphorylates, and thereby activates, Akt on a serine residue. Full Akt kinase activity is achieved when both its threonine (phosphorylated by 3-phosphoinositide dependent protein kinase [PDK1]) and serine residue are phosphorylated [62, 63]. Activated Akt in turn phosphorylates various substrates, among which are FoxO proteins [64]. Consequently, FoxO regulates expression of MAFbx and MuRF1.

## Effect on strength and body composition in resistance-trained individuals

Several trials have recently evaluated the ergogenic effects of PA in resistance-trained men. In 2012, Hoffman et al. ran a pilot study with sixteen resistance-trained men [22]. The men were randomly assigned to either ingest 750 mg PA daily or a placebo for 8 weeks. During these 8 weeks the men were instructed to follow a 4-day per week split-routine unsupervised resistance training program. Body composition (body weight, lean body mass [LBM] and body fat), strength (1-RM bench press and 1-RM squat) and ultrasonography measurements (vastus lateralis thickness and pennation angle) were made before and after the supplementation period. No significant interaction between the groups was found, although a trend (*p* = 0.065) towards a significant interaction was found for LBM change. The authors continued to statistically evaluate the results with magnitude-based inference (MBI). This indicated a likely benefit from PA for increasing 1-RM squat and a very likely benefit for increasing LBM. However, MBI results should be viewed with caution and usage of the statistical method is discouraged by some authors [65]. It should also be noted that there was essentially no change in LBM (+0.1 kg) in the placebo group, which might imply that the training stimulus was inadequate for muscle hypertrophy. Due to the small sample size, the study might have suffered from a type II error.

Following this pilot study, Joy et al. employed a similar research design in resistance-trained men (*n* = 28) [21]. The PA dosage was equal to that used by Hoffman et al. and participants followed a 3-day per week undulating resistance training program during an 8-week supplementation period. In addition, the time of PA-intake was controlled for in this study. PA was taken 30 min pre-workout (450 mg) and immediately post-workout (300 mg) on training days. On rest days, PA was taken with breakfast (450 mg) and dinner (300 mg). The PA group gained a significantly greater amount of LBM (+2.4 kg) over the 8-week period compared to the placebo group (+1.2 kg). No significant differences were observed between groups for 1-RM leg press, 1-RM bench press and fat mass change, although the latter showed a trend towards significance (*p* = 0.068). Additionally, ultrasonography revealed a significant CSA increase of the rectus femoris in the PA group compared to the placebo group.

Escalante et al. randomly assigned eighteen healthy strength-trained males to either a group consuming a PA-containing multi-ingredient supplement or a placebo for 8 weeks in conjunction with a 3-day per week undulating resistance training program [23]. The multi-ingredient supplement also contains other ingredients which can affect results, including leucine, β-hydroxy β-methylbutyrate (HMB) and vitamin D3. It is therefore completely uncertain to what extent, if any, PA affected the results of this study. However, in line with the results of Hoffman et al. and Joy et al., a significant increase in LBM change (as assessed by dual-energy X-ray absorptiometry [DXA]) was measured in the PA group compared to the placebo group. Additionally, men in the PA group significantly increased their 1-RM bench press and 1-RM leg press compared to the placebo group. Although the PA group tended to lose more fat mass than the placebo group, the result was not significant. Thigh muscle mass (as also assessed by DXA), vertical jump, push-ups to failure, pro-agility shuttle time and peak power output were also measured but did not show any significant differences between groups.

Recently, a study by Andre et al. also investigated the efficacy of lower dosages of PA (250 mg and 375 mg daily) combined with resistance training [24]. A total of 28 men was randomized to a PA group consuming 250 mg daily (PA250, *n* = 9), 375 mg daily (PA375, *n* = 9) or a placebo (PLC, *n* = 10). Similar to previous research, participants were resistance-trained and body composition, muscle size and lower-body muscle strength were determined before and after the supplementation period. However, no test for upper-body muscle strength was performed. Slightly deviating from previous research was timing of supplement intake. Whereas Joy et al. and Hoffman et al. provided PA 30 m before and directly after training, participants in this study took the supplement 60 min before training. ANOVA revealed no significant group × time interactions. Like Hoffman et al., the authors continued statistical analysis with MBI. Application of MBI showed a likely positive effect of PA on LBM and CSA of the rectus femoris compared to placebo, and a very likely positive effect on the 1-RM leg press in PA250 compared to PLC. PA375 also showed a likely positive effect on CSA of the rectus femoris, 1-RM leg press and a possible positive effect on LBM compared to PLC. However, some of the data reported in the study appear internally inconsistent, since the mean LBM change in both PA250 (+0.5 kg) and PA375 (+1.3 kg) was smaller than in PLC (+1.6 kg). This appears in conflict with the reported MBI results. Nevertheless, the lack of any significant effect as indicated by ANOVA compared to previous research might be explained by subtle differences between studies. One pronounced difference is the used dosage. Whereas the previous studies provided 750 mg PA daily to the participants, the research by Andre et al. only provided half (PA375) or one third of this (PA250).

Given the equivocal results in literature, Gonzalez et al. examined the efficacy of 750 mg of PA on muscle thickness and strength gains in resistance-trained men using a study design very similar to previous research [25]. Unfortunately, total body composition was not assessed by the authors. A total of fifteen resistance-trained men participated in the study and were instructed to follow an 8-week supervised resistance-training program with training sessions 3 days per week. Half the PA dose was taken 30 min prior to and the other half was taken directly after resistance exercise. On rest days, half the PA dose was taken with breakfast and the other half was taken with dinner. Muscle thickness of the rectus femoris, vastus lateralis, biceps brachii and triceps brachii muscles were measured via ultrasonography and 1-RM of squat, deadlift and bench press were performed as strength measures. Although all participants made improvements in each measure of muscle thickness and strength, no significant differences between the PA and placebo group were found.

Excluding the study by Escalante et al. because of supplementing PA in conjunction with other active ingredients, only the study by Joy et al. demonstrated significant improvements in lower body strength and LBM compared to placebo. Andre et al. and Hoffman et al. only found likely improvements when applying magnitude-based inference. Finally, the recent trial by Gonzalez et al. found no improvement at all in muscle thickness or in strength. It is uncertain what resulted in these differing results as there do not appear to be clear differences between studies, with the exception of the lower dosage used by Andre et al. (250 mg and 375 mg daily) in comparison to all other trials (750 mg daily). Nevertheless, small differences exist and Gonzalez et al. enumerated the following potential discrepancies: exercise supervision, resistance-training program design, dietary adherence, exercise selection for assessing maximal strength, timing of supplement ingestion, methods of assessing changes in muscle architecture and body composition and training status of study participants. It should also be noted that due to the small sample sizes used in these trials, a small effect might be easily missed.

## Conclusions

A considerable amount of research at the molecular level implicates that PA is closely involved in regulation of the mTORC1 pathway. The mTORC1 pathway is intimately involved in regulation of skeletal muscle size through regulation of muscle protein synthesis. Additionally, PA might affect muscle protein breakdown as well through regulation of FoxO, MuRF1 and MAFbx. This effect might be mediated by influencing mTORC2 activity. It would therefore be interesting to see the effect of PA supplementation on muscle mass in catabolic conditions such as cachexia or sarcopenia. However, research into the effects of PA on muscle protein breakdown is currently very limited and its practical relevance remains unknown.

A small number of studies carried out with resistance-trained men suggest that PA supplementation might be a useful dietary strategy to increase muscle mass and possibly strength in this population, although only one study has found a statistical significant effect on these parameters. This might be due to differences between studies or because of a small effect which would require larger sample sizes to consistently reach statistical significant results.

Although PA is a phospholipid present in the membranes of cells, its presence in the diet is negligible and supplementation would be required for any potential ergogenic benefits. An appropriate dosage based on the current state of research would be 750 mg supplemented daily. A lower dosage appears to be ineffective at increasing LBM or strength. An optimal time of ingestion has not yet been established. It therefore seems appropriate to recommend ingestion times in line with studies which have demonstrated benefits of the compound in athletes. Joy et al. [21] and Escalante et al. [23] showed positive effects when 450 mg PA was taken 30 m before training and 300 mg directly after. On rest days, 450 mg PA was taken with breakfast and 300 mg with dinner. Given that PA is hydrolyzed before absorption by the intestinal mucosa, after which it is re-esterified with fatty acids in the enterocytes, the fatty acid composition of the meal with which PA is taken might influence its efficacy. However, no research has evaluated this to date and it would be interesting to see if and to what extent the fatty acid composition of simultaneous meal ingestion would influence the fatty acid composition of PA reaching the bloodstream and its pharmacokinetics. Given that some research suggests that PA species containing one or two unsaturated fatty acid chains are more effective than saturated PA species in activating mTORC1, this would be of high interest. Finally, it should be noted that research investigating the safety of PA supplementation is severely lacking. Although no subjects in the discussed human trials reported side-effects, long-term data is lacking as well as more detailed safety data. Future studies aimed at collecting safety data should be conducted to fill this gap in the literature.

## Abbreviations

1-RM: 1 repetition maximum
4E-BP1: 4E binding protein 1
CDP: Cytidine diphosphate
CL: Cardiolipin
DAG: Diacylglycerol
DGK: Diacylglycerol kinase
DHAP: Dihydroxyacetone phosphate
DXA: Dual-energy X-ray absorptiometry
EDG: Endothelial differentiation gene
ER: Endoplasmic reticulum
FRB: FKBP12-rapamycin binding
G3P: Glycerol-3-phosphate
GDPH: Glycerol-3-phosphate dehydrogenase
GPAT: Glycerol-3-phosphate acyltransferase
HEK: Human embryonic kidney
HMB: β-Hydroxy β-methylbutyrate
IGF-I: Insulin-like growth factor 1
LBM: Lean body mass
LPA: Lysophosphatidic acid
LPAAT: Lysophosphatidic acid acyltransferase
MAFbx: Muscle atrophy F-box
MBI: Magnitude-based inference
mTORC1: Mechanistic target of rapamycin complex 1
MuRF1: Muscle ring finger 1
p70S6K1: P70 ribosomal protein S6 kinase 1
PA P’tase: Phosphatidic acid phosphatase
PA: Phosphatidic acid
PC: Phosphatidylcholine
PDK1: 3-phosphoinositide dependent protein kinase 1
PE: Phosphatidylethanolamine
PERK: PRK-like ER kinase
PG: Phosphatidylglycerol
PI (3) P: Phosphatidylinositol-3-phosphate
PI: Phosphatidylinositol
PI3K: Phosphoinositide-3 kinase
PIKK: PI3K-related kinase
PLA_2,_: Phospholipase A2
PLC: Phospholipase C
PLD: Phospholipase D
TNFα: Tumor necrosis factor alpha
